# Fibronectin-, vitronectin- and laminin-binding proteins at the cell walls of *Candida parapsilosis* and *Candida tropicalis* pathogenic yeasts

**DOI:** 10.1186/s12866-015-0531-4

**Published:** 2015-10-05

**Authors:** Andrzej Kozik, Justyna Karkowska-Kuleta, Dorota Zajac, Oliwia Bochenska, Sylwia Kedracka-Krok, Urszula Jankowska, Maria Rapala-Kozik

**Affiliations:** Department of Analytical Biochemistry, Faculty of Biochemistry, Biophysics and Biotechnology, Jagiellonian University in Krakow, Gronostajowa 7, 30-387 Krakow, Poland; Department of Physical Biochemistry, Faculty of Biochemistry, Biophysics and Biotechnology, Jagiellonian University in Krakow, Gronostajowa 7, 30-387 Krakow, Poland; Department of Structural Biology, Malopolska Centre of Biotechnology, Jagiellonian University in Krakow, Gronostajowa 7, 30-387 Krakow, Poland

**Keywords:** Non-albicans *Candida* species, Candidiasis, Extracellular matrix, Fibronectin, Vitronectin, Laminin

## Abstract

**Background:**

*Candida parapsilosis* and *C. tropicalis* increasingly compete with *C. albicans*—the most common fungal pathogen in humans—as causative agents of severe candidiasis in immunocompromised patients. In contrast to *C. albicans*, the pathogenic mechanisms of these two non-albicans *Candida* species are poorly understood. Adhesion of *Candida* yeast to host cells and the extracellular matrix is critical for fungal invasion of hosts.

**Methods:**

The fungal proteins involved in interactions with extracellular matrix proteins were isolated from mixtures of β-1,3-glucanase– or β-1,6-glucanase–extractable cell wall-associated proteins by use of affinity chromatography and chemical cross-linking methods, and were further identified by liquid chromatography-coupled tandem mass spectrometry.

**Results:**

In the present study, we characterized the binding of three major extracellular matrix proteins—fibronectin, vitronectin and laminin—to *C. parapsilosis* and *C. tropicalis* pseudohyphae. The major individual compounds of the fungal cell wall that bound fibronectin, vitronectin and laminin were found to comprise two groups: (1) true cell wall components similar to *C. albicans* adhesins from the Als, Hwp and Iff/Hyr families; and (2) atypical (cytoplasm-derived) surface-exposed proteins, including malate synthase, glucose-6-phosphate isomerase, 6-phosphogluconate dehydrogenase, enolase, fructose-1,6-bisphosphatase, transketolase, transaldolase and elongation factor 2.

**Discussion:**

The adhesive abilities of two investigated non-albicans *Candida* species toward extracellular matrix proteins were comparable to those of *C. albicans* suggesting an important role of this particular virulence attribute in the pathogenesis of infections caused by *C. tropicalis* and *C. parapsilosis*.

**Conclusions:**

Our results reveal new insight into host–pathogen interactions during infections by two important, recently emerging, fungal pathogens.

## Background

Several species of yeasts from the genus *Candida* can cause mild-to-severe infections that are a serious threat to the growing number of individuals with immunological impairment, such as patients subjected to surgery, transplantations, total parenteral nutrition, and insertion of catheters or stents; people after broad-spectrum antimicrobial therapy, chemotherapy, or steroid treatment [1–3]; cancer patients [4]; and neonates and the elderly [5, 6]. The epidemiological data concerning the frequency of candidiasis caused by particular species repeatedly indicate that *Candida albicans* is the most widely distributed opportunistic yeast pathogen in patients with a weakened immune system; however, other *Candida* species, including *C. glabrata*, *C. parapsilosis, C. tropicalis*, and *C. krusei*, collectively called non-albicans *Candida* (NAC) species, are increasingly emerging as causative agents of invasive mycoses [7–10]. A recent study showed that *C. albicans* and NAC species currently nearly equally contribute to the rate of invasive bloodstream candidal infections in the United States [11], whereas other reports (data of the Prospective Antifungal Therapy Alliance [PATH Alliance®] registry) suggested that the proportion of candidemia caused by NAC species even exceeds that of *C. albicans*-dependent infections (58 % vs. 42 %) [12]. Therefore, molecular studies into the pathogenesis of NAC species-dependent candidiasis should urgently be intensified, additionally because the underlying mechanisms might differ in many aspects from those well recognized for *C. albicans* [13, 14].

One important phenomenon to be considered with regard to the pathogenesis of fungal infections is the ability of the pathogen to adhere to different surfaces, including both artificial surfaces of medical devices and host cells and proteins. Generally, the first step of pathogen invasion involves the destruction of mechanical barriers that provide passive and active resistance against further microbe dissemination in the host organism. A barrier composed of a thin layer of epithelial or endothelial cells, strengthened with extracellular matrix (ECM), must be impaired during this process. During barrier impairment, an essential role is played by adhesive proteins exposed on the pathogen surface and hydrolytic enzymes secreted by the pathogen cells into the environment.

The interactions of ECM components with molecules that are exposed on the cell surface of pathogenic bacteria, also called the microbial surface components recognizing adhesive matrix molecules (MSCRAMM), have been extensively characterized [15]. *C. albicans* also exposes at the cells surface a set of adhesive proteins that interact with the major ECM proteins (ECMPs), including laminin, fibronectin, collagen type IV, and vitronectin. The ECMP-binding candidal proteins include typical glycosylphosphatidylinositol (GPI)-anchored adhesins/invasins from the agglutinin-like sequence (Als) family (i.e., Als1, Als3 and Als5) [16], as well as some proteins non-covalently bound to the cell wall, such as alcohol dehydrogenase (Adh1) [17] and glyceraldehyde 3-phosphate dehydrogenase (Tdh3) [18]. In other species from the genus *Candida*, many predicted adhesin-like proteins, potentially capable of interacting with host proteins, were indicated through a computational analysis of the genome [19]. Unfortunately, their exposure on the yeast cell surface and actual binding affinity for ECMPs have not been studied with biochemical methods, with the notable exception of the identification of a 105-kDa fibronectin-binding protein on the *C. tropicalis* cell surface [20]. The current study aimed to characterize the binding of fibronectin, laminin and vitronectin to the cell walls of *C. parapsilosis* and *C. tropicalis*—two prominent emerging fungal pathogens from the NAC group—and to identify the sets of fungal cell wall-associated proteins involved in this process.

## Methods

### Yeast strains and culturing

The *C. albicans* strain ATCC® 10231™, *C. parapsilosis* strain CDC 317 (ATCC® MYA-4646™) and *C. tropicalis* strain T1 (ATCC® MYA-3404™) were purchased from American Type Culture Collection (Manassas, VA). Cells were cultured in YPD broth (1 % yeast extract, 2 % soybean peptone and 2 % glucose) (Sigma, St. Louis, MO) at 30 °C for 16 h and then, to induce hyphae (*C. albicans*) or pseudohyphae (*C. parapsilosis* and *C. tropicalis*) formation, at 37 °C in RPMI 1640 medium (PAA Laboratories GmbH, Pasching, Austria) for 3 or 72 h.

### Proteins

Human fibronectin and vitronectin were purchased from R&D Systems (Minneapolis, MN), human laminin from Millipore (Temecula, CA), β-1,6-glucanase from Takara Bio Inc. (Otsu, Shiga, Japan), β-1,3-glucanase (lyticase) from Sigma and α1-2,3 mannosidase and α1-6 mannosidase from New England Biolabs (Ipswich, MA). Proteinase K and bovine serum albumin (BSA) were obtained from BioShop Canada Inc. (Burlington, Ontario, Canada), trypsin from Promega (Madison, WI) or Biocentrum (Krakow, Poland) and horseradish peroxidase-conjugated streptavidin solution (SA-HRP) from MP Biomedicals (Solon, OH).

### Biotinylation of ECMPs

A solution (1 mg/100 μl) of biotin N-hydroxysuccinimide ester (NHS-biotin; Sigma) in dimethylformamide was added to fibronectin, vitronectin and laminin (50 μg each) in 100 μl of 0.1 M bicarbonate buffer, pH 8.3. The mixture was incubated at 4 °C for 4 h and then dialyzed against phosphate-buffered saline (PBS) at 4 °C for 24 h. ECMPs to be used for the binding assays were biotinylated with 10 μg NHS-biotin per 50 μg protein, whereas for the chemical cross-linking experiments a 3-fold lower reagent:protein ratio was applied.

### Biotinylation of cell wall-associated proteins of filamentous forms of *Candida* spp.

To biotinylate the proteins associated with the fungal cell wall, 0.4 g (wet weight) of the *C. albicans* hyphal forms or *C. parapsilosis* and *C. tropicalis* pseudohyphal forms was washed twice with 0.1 M bicarbonate buffer, pH 8.3, suspended in 1 ml of the same buffer and treated with NHS-biotin (1 mg in 50 μl dimethylformamide) for 1 h in the dark at room temperature. Excess reagent was then removed by two washes in 50 mM phosphate buffer (pH 6.0).

### Extraction of cell wall-associated proteins from filamentous forms of *Candida* spp.

The cell wall-associated proteins were extracted from both biotinylated and non-biotinylated hyphae/pseudohyphae (see above). Isolation with β-1,3-glucanase was preceded by washing the fungal cells twice with 10 mM Tris–HCl buffer with 0.9 % NaCl, pH 7.4 and twice with 50 mM Tris–HCl buffer, pH 7.5. Next, the cells were suspended in 1 ml of the second buffer (supplemented with protease inhibitor mixture [Roche, Basel, Switzerland]) and treated with 40 mM β-mercaptoethanol and 500 U of β-1,3-glucanase for 24 h at 37 °C. To extract the proteins using β-1,6-glucanase, the cells were first washed three times with McIlvaine buffer (a mixture of 0.1 M citric acid and 0.2 M disodium phosphate), pH 6.0, with 0.5 M sodium tartrate (an osmotic stabilizer). The cells were then suspended in 1 ml of the same buffer (supplemented with protease inhibitors) and treated with 0.8 U of β-1,6-glucanase for 24 h at 37 °C.

After both types of extraction, the supernatants were collected and dialyzed against phosphate-buffered saline (PBS), pH 7.4, at 4 °C for 48 h, and the cell membrane integrity of the remaining cells was tested by staining with SYTOX® Green (Invitrogen Life Technologies, Carlsbad, CA) and Trypan Blue (Sigma). The protein concentrations in the obtained protein mixtures were determined [21] and the extracts were characterized by sodium dodecyl sulphate-polyacrylamide gel electrophoresis (SDS-PAGE) in the Laemmli system [22].

### Analysis of the binding of biotinylated fibronectin, vitronectin and laminin to filamentous forms of *Candida* spp.

Hyphal or pseudohyphal forms of *C. albicans*, *C. parapsilosis* or *C. tropicalis* which adhered to the wells of MaxiSorp 96-well microplates (Sarstedt, Nümbrecht, Germany), were obtained from 1 × 10^6^ cells propagated in RPMI 1640 medium at 37 °C for 3 h. Each step of the following binding assay was preceded by washing the fungal cells three times with PBS buffer containing 1 % BSA. The unoccupied surfaces of the microplate wells were blocked with 3 % BSA in PBS at 4 °C overnight. The wells without fungal cells (with the surface blocked with BSA) served as controls. Solutions of biotinylated fibronectin, vitronectin and laminin (50 μl) were added to the cells and incubated at 37 °C for 1.5 h with gentle shaking. The amounts of bound biotinylated proteins were determined with the use of a SA-HRP/TMB detection system as described previously [23]. The values obtained for control samples were subtracted from the total binding values.

### Binding of biotinylated fibronectin, vitronectin and laminin to *C. parapsilosis* and *C. tropicalis* pseudohyphal forms pretreated with mannosidases, proteinase K and β-1,3-glucanase

*C. parapsilosis* and *C. tropicalis* cells (5 × 10^8^) were grown in RPMI 1640 medium to obtain pseudohyphal forms and then were incubated at 37 °C for 1 h in a test-tube with 100 μl of: (1) a mixture of 64 U of α1-2,3 mannosidase and 80 U of α1-6 mannosidase in 50 mM sodium acetate buffer with 5 mM CaCl_2_, pH 5.5; (2) 0.05 mg/ml proteinase K in 20 mM Tris–HCl buffer with 20 mM NaCl, 2 mM MgCl_2_, 0.1 M DTT, 1 mM CaCl_2_, pH 8.0; or (3) 0.12 mg/ml β-1,3-glucanase and 40 mM β-mercaptoethanol in 50 mM Tris buffer, pH 7.5. The cell viability (i.e., cell membrane integrity) after these treatments was checked with SYTOX® Green. The cells were washed three times with 1 % BSA in PBS and then a 100 nM solution of biotinylated ECMP (50 μl) was added to each tube and the tubes were incubated at 37 °C for 1 h. The amount of bound labeled protein was determined as described above.

### Tests for binding of *Candida* spp. cell wall-associated proteins to ECMPs

For the saturation binding tests, fibronectin, vitronectin and laminin were immobilized in wells of MaxiSorp 96-well microplates (Sarstedt) by overnight incubation at 4 °C (5 pmol protein in total volume of 50 μl per well). After this step and all subsequent steps, the wells were washed three times with 1 % BSA in PBS. The unoccupied surfaces in each well were blocked with 3 % BSA in PBS at 4 °C (overnight). Solutions of biotinylated fungal cell wall-associated proteins (50 μl) were added to the wells and the plate was incubated at 37 °C for 1.5 h. In an alternative competitive variant of this assay, the microplate-immobilized ECMP (3 pmol fibronectin or 1.25 pmol of vitronectin or laminin in total volume of 50 μl) competed with soluble ECMP, added in increasing molar excess, for binding to biotinylated fungal cell wall-associated proteins. In both types of assay, the binding level was detected with the SA-HRP/TMB detection system.

### Affinity chromatographic isolation of fungal cell wall-associated proteins that bind fibronectin, vitronectin or laminin

Affinity gels with covalently conjugated fibronectin, vitronectin or laminin were prepared as described previously [24]. Protein immobilization proceeded in 0.1 M HEPES buffer with 80 mM CaCl_2_, pH 7.5. The β-1,3-glucanase– or β-1,6-glucanase–extracted *C. parapsilosis* or *C. tropicalis* cell wall-associated proteins (300 μg protein in 300 μl PBS, with protease inhibitors) were incubated at 37 °C for 4 h with 50 μl of the appropriate affinity gel. To remove unbound proteins, the gel was washed five times with 1 ml PBS. Bound proteins were eluted during the gel incubation with 30 μl 2 % SDS at 95 °C for 20 min. The obtained mixtures of ECMP-binding fungal proteins were separated by SDS-PAGE, with gel staining with Coomassie Brilliant Blue R-250. Control samples of the gel, not coupled to any ECMP (with reactive groups blocked with ethanolamine) but incubated with fungal proteins, were also prepared.

### Selection of fibronectin-, vitronectin- and laminin-binding fungal proteins by chemical cross-linking

Biotinylated ECMPs (20 μg in 100 μl PBS, pH 7.4) were incubated in the dark at 4 °C for 2 h with 0.5 mM sulfosuccinimidyl 2-([4,4′-azipentanamido]ethyl)-1,3′-dithiopropionate (sulfo-SDAD) (Thermo Fisher Scientific Inc., Woltham, MA). The reaction was stopped with 50 mM Tris for 15 min and the excess reagent was removed by dialysis against PBS at 4 °C overnight in the dark. The particular labeled ECMP was incubated with a mixture of fungal cell wall-associated proteins (300 μg in 300 μl PBS) at 37 °C for 1 h in the dark. The samples were placed on ice and exposed to UV radiation (365 nm) for 15 min. Covalently linked pairs of biotinylated ECMP–fungal protein were adsorbed on MagnaBind Streptavidin Beads (Thermo Fisher Scientific Inc.). The beads were washed five times with 1 ml PBS, with intense stirring, to remove unbound, unlabeled proteins. The fungal proteins were dissociated from the beads by boiling for 30 min in 30 μl 2.5 % β-mercaptoethanol and 2 % SDS. These proteins were separated by SDS-PAGE, and protein bands were visualized by staining with Coomassie Brilliant Blue R-250.

### Protein identification by mass spectrometry

To identify the proteins in the stained bands after SDS-PAGE, gel pieces were manually excised, destained at 37 °C by several washes in 25 % and 50 % acetonitrile, reduced with 50 mM dithiothreitol at 56 °C for 45 min and alkylated with 55 mM iodoacetamide at room temperature for 2 h in the dark. Residual reagents were removed with 50 % acetonitrile in 25 mM ammonium bicarbonate buffer (NH_4_HCO_3_). Gel pieces were dehydrated in 100 % acetonitrile and dried for 15 min using a SpeedVac. Next, 15 μl of trypsin solution (10 ng/μl in 25 mM NH_4_HCO_3_, pH 8.0) was added for 15 min and an additional 20 μl of 25 mM NH_4_HCO_3_ was then added. The digestion was performed at 37 °C overnight. Peptides were extracted by sonication and drying with 100 % acetonitrile. The extracts were evaporated to dryness and resuspended in 2 % acetonitrile with 0.05 % trifluoroacetic acid or 10 % acetonitrile with 0.1 % formic acid.

Two methods were used for peptide analysis by liquid chromatography-coupled tandem mass spectrometry (LC-MS/MS). One method used an UltiMate 3000 RSLCnano System (Dionex, Carlsbad, CA), coupled to a micrOTOF-QII mass spectrometer (Bruker, Bremen, Germany), containing an Apollo Source ESI nano-sprayer equipped with low-flow nebulizer. The peptide mixtures were injected on a C18 precolumn (Acclaim PepMap Nano Trap Column) using 2 % acetonitrile with 0.05 % formic acid as a mobile phase and further separated on a 15 cm × 75 μm reversed-phase column (Acclaim PepMap 75 μm 100 Å Nano Series TM Column) using a gradient of 2–40 % acetonitrile with 0.05 % trifluoroacetic acid in 30 min. The mass spectrometer was operated in a standard data-dependent acquisition MS/MS mode, with fragmentation of the most intensive precursor ions.

The second method for LS-MS/MS analysis used a HCT Ultra ion-trap mass spectrometer equipped with an electrospray ionization ion source and electron transfer dissociation II fragmentation module (Bruker, Bremen, Germany) and coupled to an ultrahigh-performance liquid chromatography Dionex Ultimate 3000 system. The peptides were separated on a 100 mm × 2.1 mm Accucore C18 column (particle size, 2.6 μm) (Thermo Fisher Scientific), with a gradient of 10–70 % of 0.1 % formic acid in 80 % acetonitrile in 38 min. The mass spectrometer was operated in a standard MS/MS mode with simultaneous fragmentation of the most intensive precursor ions by collision-induced dissociation and electron transfer dissociation.

Measured MS spectra were recalibrated using fragment ions of trypsin-derived peptides.

Mascot Generic format (.mgf) files were generated by pre-processing the raw data with Data Analysis 4.0 software (Bruker). The lists of peaks obtained were searched against the nonredundant protein database of the NCBI (all entries: 53,183,920 sequences) or with taxonomy restriction (Fungi: 3,246,022 sequences) using an in-house Mascot server (v.2.3.0; Matrix Science, London, UK). The following search parameters were applied: enzyme specificity – trypsin; permitted number of missed cleavages – 1; fixed modification – carbamidomethylation (C); variable modifications – oxidation (M); protein mass – unrestricted; peptide mass tolerance of ± 20 ppm or ± 0.3 Da and fragment mass tolerance of ± 0.05 Da or ± 0.3 Da, for the two LC-MS/MS systems specified above, respectively.

### Ethics statement

No ethical approval was required for this study since it did not involve humans, human data or animals.

## Results

### General characteristics of the binding of biotinylated fibronectin, vitronectin and laminin by filamentous forms of *Candida* spp.

**Fig. 1:**
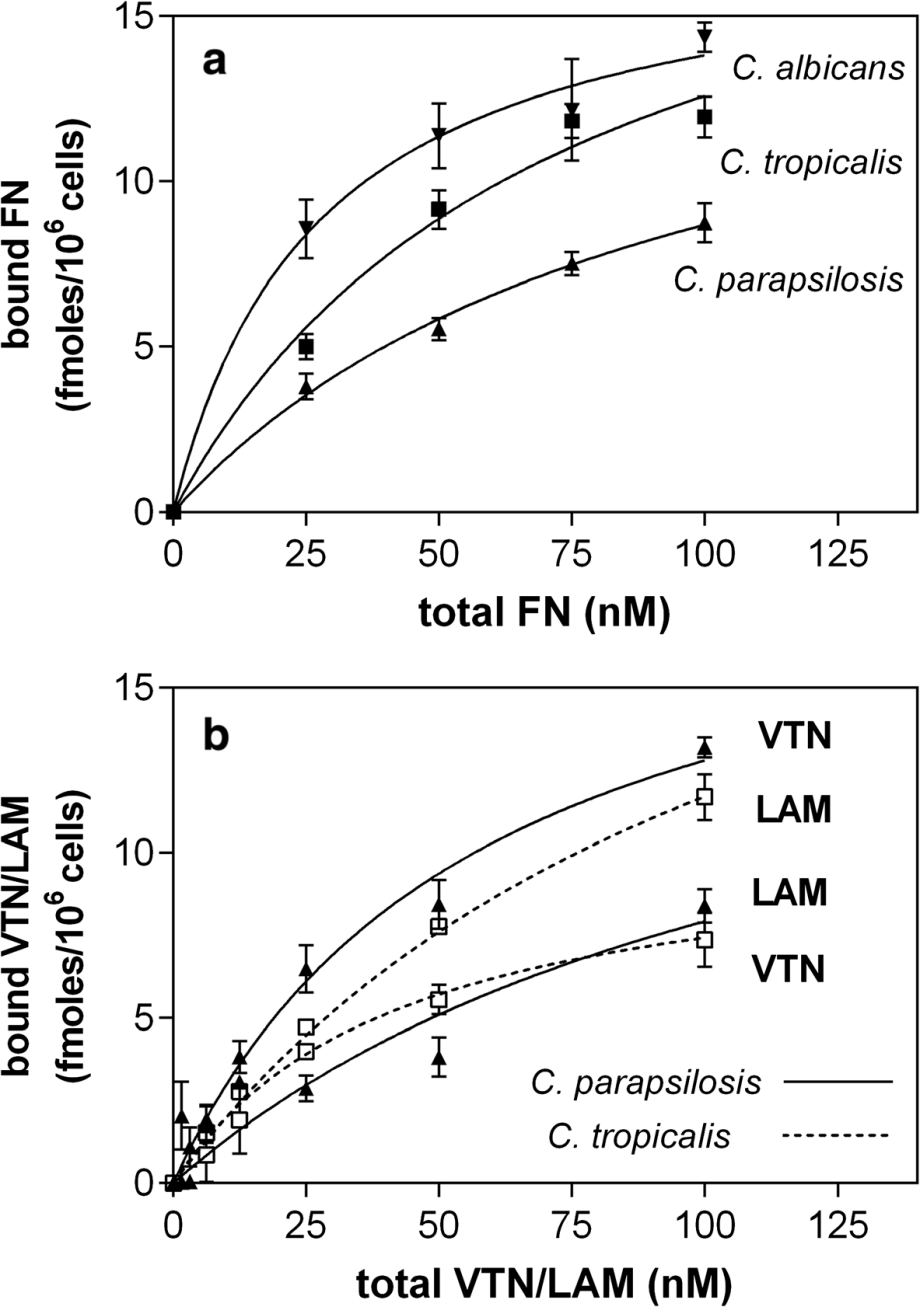
Binding of extracellular matrix proteins by *Candida* spp. filamentous forms. **a** Fibronectin (FN) binding by *C. albicans*, C*. parapsilosis* and *C. tropicalis*; **b** vitronectin (VTN) and laminin (LAM) binding by *C. parapsilosis* and *C. tropicalis*. Filamentous forms obtained from 1 × 10^6^ yeast cells and adsorbed in wells of MaxiSorp microplates were incubated at 37 °C for 1.5 h with 50 μl of biotin-labeled FN, VTN or LAM solutions. Additional wells without adsorbed fungal cells but with BSA-blocked surfaces served as controls. The amounts of bound protein were determined using an SA-HRP/TMB system. The readings from control wells were subtracted from the total binding values. Representative binding plots are presented, in which data points represent means from three determinations (three independent wells) ± standard deviation

Representative plots for saturable binding of biotin-labeled fibronectin by *C. albicans* hyphae and *C. parapsilosis* and *C. tropicalis* pseudohyphae are shown in Fig. 1a. The binding levels were within a range of 8–20 fmoles for hyphae/pseudohyphae generated from 1 × 10^6^ cells, and decreased in the order of *C. albicans* > *C. tropicalis* > *C. parapsilosis*. The binding of vitronectin and laminin by *C. parapsilosis* and *C. tropicalis* pseudohyphae is shown in Fig. 1b. For each of the latter species, the binding capacities for fibronectin and laminin were comparable; for vitronectin, they were slightly higher in *C. parapsilosis* and lower in *C. tropicalis*. The highest (>2-fold) difference in binding capacity was noted between the two species for vitronectin.

**Fig. 2:**
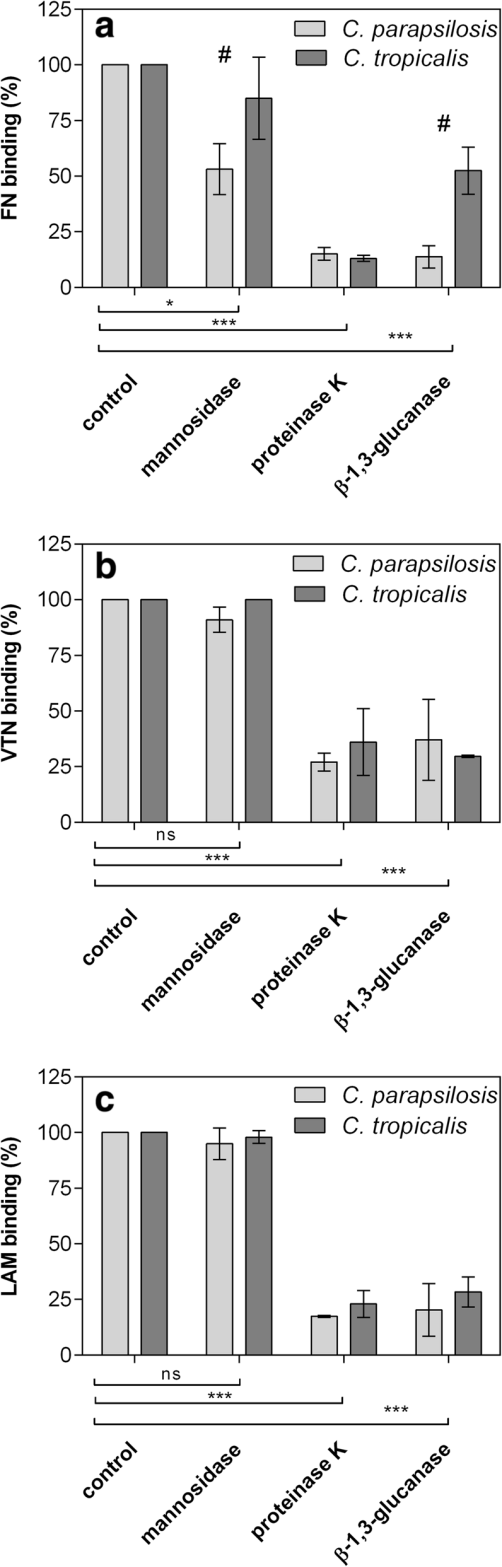
Involvement of the major *C. parapsilosis* and *C. tropicalis* cell wall components in the binding of ECMPs. Binding of biotinylated fibronectin (FN) (**a**), vitronectin (VTN) (**b**) and laminin (LAM) (**c**) was determined for *C. parapsilosis* and *C. tropicalis* pseudohyphae (obtained from 5 × 10^8^ yeast cells) that were pretreated with enzymes that digest the major constituents of the fungal cell wall, namely: (1) a mixture of α1-2,3 mannosidase and α1-6 mannosidase; (2) proteinase K; and (3) β-1,3-glucanase. The cell suspensions were then incubated with 100 nM of each labeled ECMP for 1.5 h at 37 °C. The amount of bound protein was determined using the SA-HRP/TMB detection system. The binding level of the control (untreated) cells was considered 100 %. Bars represent the mean values of three determinations (three independent yeast cultures) ± standard deviation. Statistical significance levels against the control are indicated with *p < 0.05, ***p < 0.001 or ns “not significant”, whereas statistically significant comparisons between two tested species are marked with # (*p* < 0.05)

Involvement of major classes of fungal cell wall components, namely, cell wall-associated proteins, glucans and mannans, was tested by determination of the binding of each ECMP to pseudohyphae that were pretreated with: (1) proteinase K, which digests proteins exposed on the surface of fungal cells; (2) β-1,3-glucanase, which hydrolyzes the β-1,3-glucan network and also releases a set of β-1,3-glucan–associated proteins; and (3) a mixture of mannosidases, which degrade the mannan layer of the cell wall. As shown in Fig. 2, the cell wall-associated proteins play a predominant role in ECMP binding in both *Candida* species investigated. Of other cell wall components, apart from proteins, mannans were additionally found to be important for the binding of fibronectin, as judged from a decrease of the binding level by about 40 % and 20 % for C. *parapsilosis* and *C. tropicalis*, respectively, after mannosidase treatment. A statistically significant difference between the effect of β-1,3-glucanase treatment on fibronectin binding in *C. parapsilosis* and *C. tropicalis* suggests a lower contribution of β-1,3-glucan to the interactions of *C. tropicalis* cell wall with this ECMP, relatively to the other species.

**Fig. 3:**
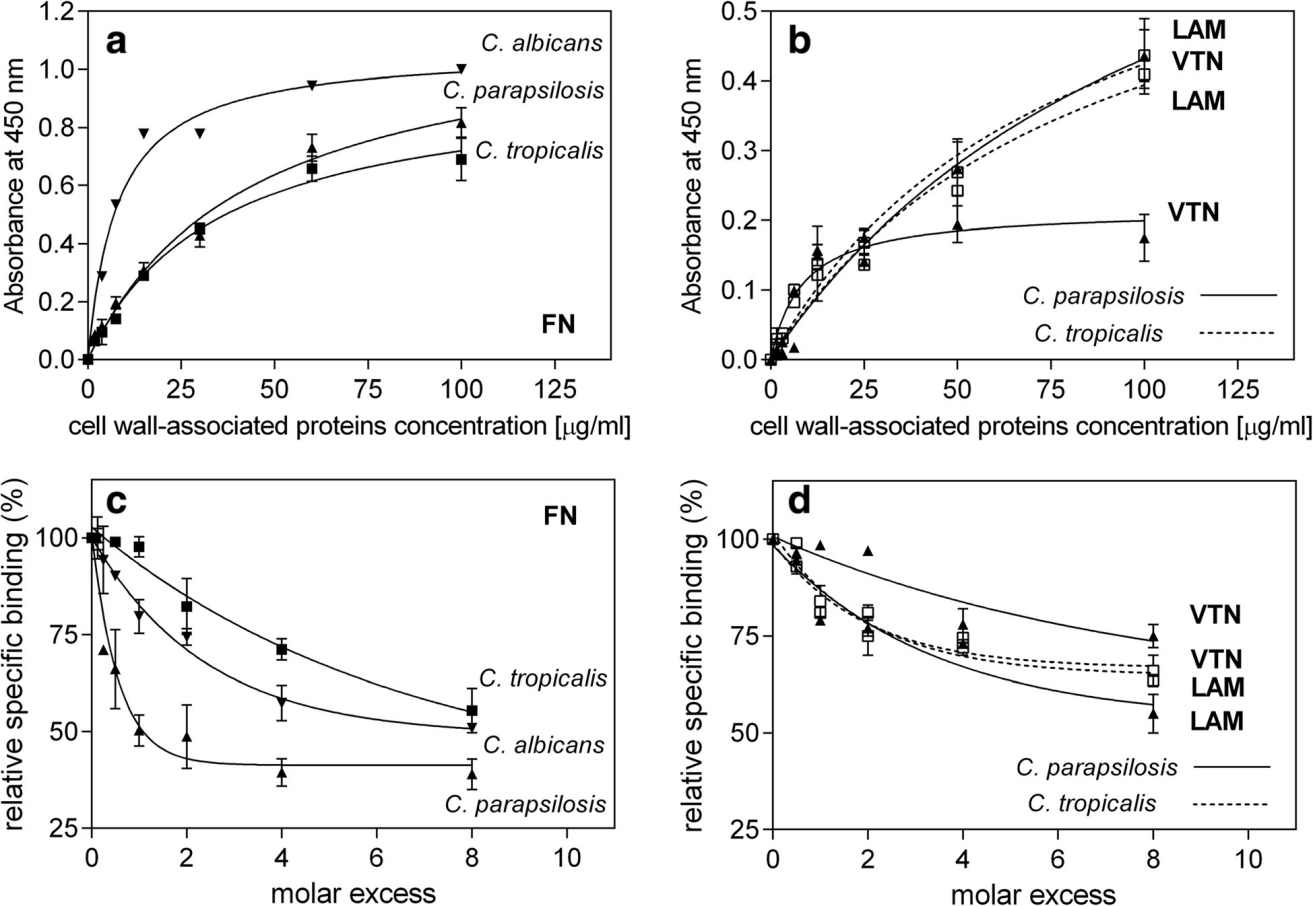
Interactions of *Candida* spp. cell wall-associated proteins with fibronectin (FN), vitronectin (VTN) and laminin (LAM). In panels **a** and **b**, the plots are presented for saturable binding of biotinylated cell wall-associated proteins extracted from filamentous forms of *C. albicans*, *C. parapsilosis* and *C. tropicalis* to immobilized FN (**a**) and of cell wall-associated proteins of *C. parapsilosis* and *C. tropicalis* to immobilized VTN and LAM (**b**) (5 pmoles of FN, VTN or LAM adsorbed into wells of MaxiSorp microplates with the unoccupied surfaces blocked with BSA). Panels **c** and **d** show the respective plots for the displacement of biotinylated cell wall-associated proteins (40 μg/ml for FN binding or 15 μg/ml for VTN and LAM binding) from microplate-immobilized ECMPs (3 pmoles of FN or 1.25 pmoles of VTN or LAM per well) by soluble FN, VTN and LAM added at increasing concentrations. Wells without immobilized ECMPs but coated with BSA served as controls and the values obtained for those wells were subtracted from the total binding values. Results from representative experiments are presented, in which data points represent mean values from three determinations (three wells) ± standard deviation

Further experiments were thus focused on the proteinaceous components of the fungal cell wall. These were extracted from pseudohyphal forms of the two investigated NAC species and, for comparative purposes, from *C. albicans* hyphae, by a procedure that is postulated to protect the native binding activities toward biological ligands. Therefore, strong denaturants or otherwise harsh conditions could not be applied, which inevitably limited the yield of the protein extraction process. Of the two glucanases used, β-1,3-glucanase was employed to hydrolyze the entire β-1,3-glucan network to release all proteins connected with or embedded in this structure while β-1,6-glucanase was used to cleave the anchors through which the main adhesins are known to be covalently bound. After these treatments, more than 95 % of the cells remained viable, excluding the possibility of a significant contamination of the obtained protein mixtures with cytoplasmic proteins. The obtained cell wall-associated proteins possessed binding activity toward fibronectin, vitronectin and laminin, as determined using two kinds of binding tests: (1) saturable binding of biotinylated ECMPs (Fig. 3a, b), a test similar to that used for the whole cells and presented in Fig. 1 above; and (2) a competitive test based on the displacement of the biotinylated cell wall-associated proteins from microplate-immobilized fibronectin, vitronectin or laminin by soluble ECMPs (Fig. 3c, d). Notably, the highest displacement (in a range of 50–60 % at 10-fold molar excess of soluble protein) was observed for fibronectin. Thus, the binding of vitronectin and laminin might be considered to be less “specific”, particularly for *C. parapsilosis*.

### Identification of *C. parapsilosis* and *C. tropicalis* cell wall-associated proteins that bind fibronectin, vitronectin and laminin

Subsets of ECMP-binding proteins were isolated from the whole mixtures of cell wall-associated proteins by using two methods: (1) affinity chromatography on agarose beads with immobilized fibronectin, vitronectin and laminin; and (2) fixing, by chemical cross-linking, the protein–protein complexes that were pre-formed in solution. With the first method [24], the mixture of cell wall-associated proteins was incubated with affinity beads and, following removal of the unbound proteins by washing, the mixture was boiled in 2 % SDS to elute adsorbed ECMP-binding fungal proteins and subjected to SDS-PAGE. Only those protein bands that were not visible in the electrophoretic pattern of a control sample, eluted from the gel without immobilized ECMP (instead the reactive groups were blocked with ethanolamine), were further analyzed by nano LC-MS/MS.

With the second method, low-biotinylated fibronectin, vitronectin and laminin were modified in the dark with a bifunctional reagent (sulfo-SDAD) that at one end contains an amine-reactive NHS-ester group. The modified ECMPs were then allowed to interact with the whole mixtures of fungal cell wall-associated proteins. The mixture was then illuminated to activate the second head of the cross-linker, and the covalently fixed ECMP–fungal protein complexes were adsorbed on streptavidin-conjugated beads. The elution of adsorbed proteins was performed by boiling beads in the presence of a reducing agent in order to cleave the disulfide bond localized within the cross-linker arm. This procedure was assumed to release only fungal proteins, and these were further subjected to SDS-PAGE separation and LC-MS/MS analysis. A portion of beads that was incubated with unlabeled fungal proteins that were not subjected to cross-linking but eluted in the same conditions served as a control. As with the first method, only those protein bands that did not appear in the control sample were analyzed.

A list of identified fibronectin-, vitronectin- and laminin-binding fungal proteins, with database accession numbers, protein names, theoretical molecular masses, mass spectrometric analysis parameters and the specifications of the procedures applied, is presented in Table 1.

**Table 1:** Mass spectrometric identification of fibronectin-, vitronectin- and laminin-binding *C. parapsilosis* and *C. tropicalis* cell wall-associated proteins

*C. parapsilosis*									
NCBI protein database accession number	Protein	Molecular mass [kDa]	Scores	Number of peptides	Sequence coverage [%]	Method of extraction from fungal cell wall		Method of identification	
						β-1,3-glucanase	β-1,6-glucanase	AFC	CL
Fibronectin-binding proteins									
gi|354547941	hypothetical protein CPAR2_404800 [*C. parapsilosis*], cell wall agglutinin, N-terminal, ligand sugar binding	143.6	736	13	14		+	**+**	
gi|354547939	hypothetical protein CPAR2_404780 [*C. parapsilosis*], cell wall agglutinin, N-terminal, ligand sugar binding	113.6	525	10	13		**+**	**+**	
gi|354546787	hypothetical protein CPAR2_211630 [*C. parapsilosis*], similar to *C. albicans* Eft2, elongation factor 2	93.8	259	11	14	**+**	**+**	**+**	**+**
gi|354547813	hypothetical protein CPAR2_403510 [*C. parapsilosis*], similar to *C. albicans* Rbt1, cell wall protein with similarity to Hwp1, required for virulence	83.8	73	2	1		**+**		**+**
gi|354545198	hypothetical protein CPAR2_804740 [*C. parapsilosis*], similar to *C. albicans* Tkl1, putative transketolase	74.6	166	6	8	**+**	**+**	**+**	**+**
gi|354545113	hypothetical protein CPAR2_803890 [*C. parapsilosis*], similar to *C. albicans* Mls1, malate synthase	62.7	1434	32	72	**+**	**+**	**+**	**+**
gi|354544475	hypothetical protein CPAR2_301880 [*C. parapsilosis*], putative amidase	61.7	938	18	38	**+**	**+**	**+**	**+**
gi|354546116	hypothetical protein CPAR2_204880 [*C. parapsilosis*], similar to *C. albicans* Pgi1, putative glucose-6-phosphate isomerase	61.5	104	3	7		**+**	**+**	
gi|354545980	hypothetical protein CPAR2_203520 [*C. parapsilosis*], similar to *C. albicans* Gnd1, putative 6-phosphogluconate dehydrogenase	55.4	184	5	15	**+**	**+**		**+**
gi|354546805	hypothetical protein CPAR2_211810 [*C. parapsilosis*], similar to *C. albicans* Gpm1, phosphoglycerate mutase	27.6	366	13	33		**+**		**+**
Vitronectin-binding proteins									
gi|8927048	elongation factor 2 [*C. parapsilosis*]	90.3	362	8	10	**+**	**+**	**+**	**+**
gi|354545198	hypothetical protein CPAR2_804740 [*C. parapsilosis*], similar to *C. albicans* Tkl1, putative transketolase	74.6	146	3	4	**+**	**+**	**+**	**+**
gi|354545113	hypothetical protein CPAR2_803890 [*C. parapsilosis*], similar to *C. albicans* Mls1, malate synthase	62.7	927	19	43		**+**	**+**	**+**
gi|354544475	hypothetical protein CPAR2_301880 [*C. parapsilosis*], putative amidase	62.1	1452	25	45	**+**	**+**	**+**	**+**
gi|354546116	hypothetical protein CPAR2_204880 [*C. parapsilosis*], similar to *C. albicans* Pgi1, putative glucose-6-phosphate isomerase	61.5	655	15	22		**+**	**+**	
gi|354545980	hypothetical protein CPAR2_203520 [*C. parapsilosis*], similar to *C. albicans* Gnd1, putative 6-phosphogluconate dehydrogenase	55.4	636	14	31	**+**	**+**	**+**	**+**
gi|354546348	hypothetical protein CPAR2_207210 [*C. parapsilosis*], similar to *C. albicans* Eno1, enolase	47	229	4	13		**+**	**+**	
gi|354545888	hypothetical protein CPAR2_202600 [*C. parapsilosis*], similar to *C. albicans* Tal1, putative transaldolase	35.7	526	10	36		**+**	**+**	
Laminin-binding proteins									
gi|354547939	hypothetical protein CPAR2_404780 [*C. parapsilosis*], cell wall agglutinin, N-terminal, ligand sugar binding	113.6	93	3	2		**+**	**+**	
gi|354545113	hypothetical protein CPAR2_803890 [*C. parapsilosis*], similar to *C. albicans* Mls1, malate synthase	62.7	425	9	20		**+**		**+**
gi|354544475	hypothetical protein CPAR2_301880 [*C. parapsilosis*], putative amidase	61.7	1629	67	58	**+**	**+**		**+**
gi|354546805	hypothetical protein CPAR2_211810 [*C. parapsilosis*], similar to *C. albicans* Gpm1, phosphoglycerate mutase	27.3	167	4	18	**+**			**+**
Fibronectin-binding proteins									
gi|255722852	predicted protein [*C. tropicalis* MYA-3404], hyphally regulated cell wall protein, N-terminal	194.6	202	6	4		+	+	
gi|255728333	malate synthase [*C. tropicalis* MYA-3404]	62.4	921	23	58		+	+	+
gi|255727881	enolase 1 [*C. tropicalis* MYA-3404]	46.9	619	33	55		+	+	+
gi|255732698	fructose-1,6-bisphosphatase [*C. tropicalis* MYA-3404]	40.5	134	4	14	+	+	+	+
gi|255722021	transaldolase [*C. tropicalis* MYA-3404]	35.5	123	3	8		+	+	
Vitronectin-binding proteins									
gi|255722852	predicted protein [*C. tropicalis* MYA-3404], hyphally regulated cell wall protein, N-terminal	194.6	107	3	2		+	+	
gi|240134900	elongation factor 2 [*Candida tropicalis* MYA-3404]	90.2	378	10	11		+	+	
gi|255728333	malate synthase [*C. tropicalis* MYA-3404]	62.4	529	12	25		+		+
gi|255727881	enolase 1 [*C. tropicalis* MYA-3404]	46.9	157	5	18	+		+	
gi|255732698	fructose-1,6-bisphosphatase [*C. tropicalis* MYA-3404]	40.5	320	7	25	+	+	+	+
gi|255722021	transaldolase [*C. tropicalis* MYA-3404]	35.5	513	10	28		+		+
Laminin-binding proteins									
gi|255722852	predicted protein [*C. tropicalis* MYA-3404], hyphally regulated cell wall protein, N-terminal	194.6	103	2	1		+	+	
gi|240134900	elongation factor 2 [*Candida tropicalis* MYA-3404]	90.2	259	7	8		+	+	
gi|255728333	malate synthase [*C. tropicalis* MYA-3404]	62.4	337	10	22		+	+	+
gi|240132975	peroxisomal catalase [*Candida tropicalis* MYA-3404]	55.1	104	3	5		+	+	
gi|255727881	enolase 1 [*C. tropicalis* MYA-3404]	46.9	189	4	12		+	+	
gi|255732698	fructose-1,6-bisphosphatase [*C. tropicalis* MYA-3404]	40.5	136	4	15	+		+	
gi|255722021	transaldolase [*C. tropicalis* MYA-3404]	35.5	487	12	44		+	+	

Cell wall-associated proteins were extracted from *C. parapsilosis* and *C. tropicalis* pseudohyphae, using either β-1,3-glucanase (+β-mercaptoethanol) or β-1,6-glucanase. Subsets of ECMP-binding proteins were isolated from the whole cell wall protein mixtures, using affinity chromatography (AFC) or cross-linking (CL) methods. After SDS-PAGE electrophoresis, specific protein bands were excised and digested using trypsin. Peptides were analyzed using the UltiMate 3000 RSLCnano System coupled to a micrOTOF-QII mass spectrometer and Apollo Source ESI nano-sprayer (for AFC-isolated proteins) or an Dionex Ultimate 3000 UHPLC system coupled to an HCT Ultra ETDII mass spectrometer equipped with an ESI ion source (for CL-isolated proteins). The obtained lists of peaks were searched against the NCBI protein database using an in-house Mascot server. The data represent a combination of results obtained for the extracts of cell wall-associated proteins, prepared from three independent yeast cultures. For a given extraction method (β-1,3-glucanase vs. β-1,6-glucanase) and ECMP-binder isolation method (AFC vs. CL), only those proteins are listed that were found on the analysis of all three yeast cultures. Hence, each “+” sign means that a given protein was found at least three times. The best Mascot parameters (scores, number of identified peptides and sequence coverage) ever recorded for a given protein are specified; the proteins identified with a score > 70 and a number of identified peptides ≥ 2 are only listed

In *C. parapsilosis*, 10 fibronectin-binding proteins were found. Half of them were identified with both methods used for the extraction of cell wall-associated proteins. These fibronectin-binding compounds included proteins similar to *C. albicans* elongation factor 2, transketolase, malate synthase, putative amidase and 6-phosphogluconate dehydrogenase. Extraction with β-1,6-glucanase revealed two proteins (CPAR2_404800 and CPAR2_404780) with N-terminal sequence similarity to the *C. albicans* Als protein family and a protein with similarity to *C. albicans* Rbt1, a protein that is similar to hyphal wall protein 1 (Hwp1). All three proteins are equipped with C-terminal GPI anchors. Additionally, proteins similar to *C. albicans* glucose-6-phosphate isomerase and phosphoglycerate mutase were identified as putative *C. parapsilosis* fibronectin-binding proteins. The first was also identified as a vitronectin-binding protein and the second as a laminin-binding protein in *C. parapsilosis*. Moreover, the other three laminin-binding proteins found, including one protein with an N-terminal similar to that of Als, were the same as those identified during the fibronectin-binding analysis. In a set of putative *C. parapsilosis* vitronectin-binding proteins, except the homolog of *C. albicans* glucose-6-phosphate isomerase, other five proteins were also able to bind fibronectin.

In *C. tropicalis*, four proteins, including malate synthase (detected only after the β-1,6-glucanase extraction), fructose-1,6-bisphosphatase, enolase and transaldolase were common fibronectin, vitronectin and laminin binders. Elongation factor 2 was additionally identified as a laminin- and vitronectin-binding protein, while catalase as a laminin-binding protein. In fibronectin-, vitronectin- and laminin-binding analyses, a putative GPI-anchored protein with N-terminal sequence similarity to *C. albicans* hyphally regulated cell wall protein (Hyr) was identified after extraction from the *C. tropicalis* cell wall with β-1,6-glucanase.

## Discussion

The ECM is a connective tissue that not only provides a mechanical support for cells nearby but is also involved in their migration, adherence and proliferation. It is composed of glycosaminoglycans, proteoglycans and fibrous proteins [25]. Fibronectin, one of the major proteinaceous components of the ECM, is a large glycoprotein with a molecular mass of about 440 kDa. Fibronectin isoforms occur in both plasma and ECM. In the ECM, this protein is responsible for anchoring the cells to the ECM via binding to cell membrane integrins. Vitronectin is a smaller, 75-kDa protein found in plasma that also forms bridges between the cells and the ECM. Laminin—the main component of the ECM basement membrane—also binds to cell surfaces and self-associates to form a flexible network that provides a scaffold for the cell layer. The RGD domains (named after their critical three-amino acid sequence of Arg-Gly-Asp) are crucial for the interaction of fibronectin, vitronectin and laminin with cells [26]. All of these three proteins can interact with other components of the ECM to enhance its stability, elasticity and strength [25]. For bacteria and other pathogenic microorganisms, adhesion to the ECM and human host cells is a critical step in the first stage of infection [15]. This phenomenon is also essential for development of the commensal status of microbes—such as the *Candida* yeasts—that belong to the physiological microbiota of humans.

The strong binding of fibronectin, laminin and vitronectin by *C. albicans* cells involves multiple cell wall proteins [16–18, 27]. Fibronectin- and laminin-binding by *C. tropicalis* has also been reported [20], albeit without deeper insight into this phenomenon. Significantly, the adhesion of these two ECMPs to *C. tropicalis* cells was significantly higher than to *C. albicans* [28]. In our current analyses, for the first time, the *C. parapsilosis* and *C. tropicalis* cell wall-associated proteins were characterized in terms of interactions with fibronectin, vitronectin and laminin. These two NAC species are characterized by high adhesiveness and an ability to form biofilm and are currently emerging fungal pathogens with increasing rates of morbidity and mortality [2, 29].

The candidal cell wall is composed of proteins, chitin, β-1,3-glucan and β-1,6-glucan, mannans and small amounts of lipids [30, 31]. Our studies indicated that cell wall-associated proteins are crucial to the binding of fibronectin, vitronectin and laminin by *C. parapsilosis* and *C. tropicalis* pseudohyphae, although a smaller contribution from polysaccharides cannot be excluded. Complementary data were previously presented for *C. albicans* [32, 33], considered to be the most adhesive and accordingly also the most pathogenic species of the genus *Candida* [34, 35]. Our current observation that the adhesive abilities of two investigated NAC species toward ECMPs were comparable to those of *C. albicans* suggests an important role of this particular virulence attribute in the pathogenesis of infections caused by *C. tropicalis* and *C. parapsilosis*.

The protein mixtures, extracted from the fungal cell wall with the use of β-1,3-glucanase and β-1,6-glucanase, were confirmed to possess high fibronectin-, vitronectin- and laminin-binding activity. Hence, the cell wall-associated ECMP-binding components underwent the isolation by affinity chromatography and chemical cross-linking, and were further identified by mass spectrometry. The results obtained by these two different identification methods and presented in Table 1 seemed to be complementary and indicated proteins with distinct affinities for fibronectin-, vitronectin- or laminin as well as some that shared an ability to bind two or three ECMPs. For comparative purposes, a list of ECMP-binding proteins, unequivocally identified in *C. albicans* in the numerous previous studies, is presented in Table 2, and will be used in the further discussion below.

**Table 2:** *Candida albicans* proteins with the previously confirmed binding affinity to human fibronectin, vitronectin and laminin

Abbreviation	Protein name	Method used for protein identification and confirmation of binding to ECMPs	Reference
Fibronectin-binding proteins			
Als1	Agglutinin-like sequence protein 1	expression in S*accharomyces cerevisiae* followed by binding to magnetic beads coated with fibronectin	[38]
		expression in *S. cerevisiae* followed by binding to microplate-immobilized fibronectin	[16]
		expression of the Als1 N-terminal part in *S. cerevisiae* followed by its purification and surface plasmon resonance measurements	[39]
Als3	Agglutinin-like sequence protein 3	expression in *S. cerevisiae* followed by binding to microplate-immobilized fibronectin	[16]
		expression in *S. cerevisiae* followed by microtiter plate-based assay	[40]
Als5	Agglutinin-like sequence protein 5	expression in *S. cerevisiae* followed by binding to microplate-immobilized fibronectin	[16]
		binding of protein fragments—Als5 1–431 and Als5 1-664—to fibronectin-coated microtiter plate wells	[41]
Hwp1	Hyphal cell wall protein 1	expression in *S. cerevisiae* followed by microtiter plate-based assay	[40]
Tdh3	Glyceraldedyhde-3-phosphate dehydrogenase	immunoaffinity purification of Tdh3 followed by ligand Western Blot assay	[18]
Adh1	Alcohol dehydrogenase	screening of *C. albicans* cDNA library with polyclonal antibodies against receptor for fibronectin	[17]
Vitronectin-binding proteins			
Adh1	Alcohol dehydrogenase	screening of *C. albicans* cDNA library with polyclonal antibodies against receptor for vitronectin	[17]
Gpm1	Phosphoglycerate mutase	enzyme-linked ligand sorbent assay (ELISA) with microplate-immobilized recombinant Gpm1	[45]
Laminin-binding proteins			
Als1	Agglutinin-like sequence protein 1	expression in S. *cerevisiae* followed by binding to magnetic beads coated with laminin	[38]
		expression in *S. cerevisiae* followed by binding to microplate-immobilized laminin	[16]
		expression of the Als1 N-terminal part in *S. cerevisiae* followed by its purification and surface plasmon resonance measurements	[39]
Als3	Agglutinin-like sequence protein 3	expression in *S. cerevisiae* followed by binding to microplate-immobilized laminin	[16]
Als5	Agglutinin-like sequence protein 5	expression in *S. cerevisiae* followed by binding to microplate-immobilized laminin	[16]
Tdh3	Glyceraldedyhde-3-phosphate dehydrogenase	immunoaffinity purification of Tdh3 followed by ligand Western Blot assay	[18]

As in *C. albicans*, the fibronectin-, vitronectin- and laminin-binding proteins of *C. parapsilosis* and *C. tropicalis* belong to two groups: (1) true cell wall components similar to typical *C. albicans* adhesins; and (2) atypical, cytoplasm-derived but surface-exposed proteins, sometimes collectively termed “moonlighting proteins” [36]. Evidence suggests that gene families coding for specialized adhesins in *C. albicans* also exist in the *C. parapsilosis* and *C. tropicalis* genome [19] but to date their protein products have not been characterized in detail at the molecular or functional level [37]. In our current study, at least two putative representatives of the Als-like protein family and a putative homolog of *C. albicans* Rbt1 protein from the Hwp family were found to possess the ability to bind ECMPs in *C. parapsilosis*. In *C. albicans* (Table 2) three representatives of Als-like protein family—Als1, Als3 and Als5—were indicated as proteins capable of fibronectin and laminin binding and, additionally, also Hwp1 was found to be a fibronectin-binding protein [16, 38–41]. In *C. tropicalis*, an ECMP-binding protein with high sequence similarity to members of *C. albicans* adhesins from the Iff/Hyr (hyphally regulated) protein family was identified. The *C. albicans* homologs of adhesive proteins are expected to mainly be covalently attached to the layer of β-1,6-glucans through their GPI anchor, or, as in the case of some members of the Iff family, might be bound by alkali-labile and disulfide bonds to the glucan network or other cell wall proteins [37, 42, 43].

Moreover, several atypical proteins that are probably more loosely attached to the *C. parapsilosis* and *C. tropicalis* cell wall were identified as putative ECMP binding partners. Involvement of some of these “moonlighting” proteins, such as enolase, phosphogluconate mutase, elongation factor 2 and 6-phosphogluconate dehydrogenase, in ECMP binding is additionally supported by previous reports that their homologs, present in the *C. albicans* cell wall, were able to bind the human high-molecular weight kininogen [24] and that the homologs of the first two “moonlighting” proteins had an affinity to human plasminogen [44]. Additionally, phosphogluconate mutase was indicated as *C. albicans* cell surface-exposed protein involved in interactions with vitronectin [45]. Moreover, recent studies of surface antigens of NAC species indicated that both *C. parapsilosis* and *C. tropicalis* possess cell wall-associated enolases capable of triggering the host immune response during fungal infection [46, 47]. Thus, they are likely to play a role in other pathogenicity-associated processes, such as adhesion to host cells and proteins [36]. It should also be emphasized that enolase is a confirmed cell surface-exposed plasminogen-binding protein in many different organisms [48].

In case of *C. albicans*, two further atypical proteins were found as ECMP-binding proteins, namely alcohol dehydrogenase [17] and glyceraldehyde-3-phosphate dehydrogenase [18]. Neither was identified as engaged in ECMP-binding phenomenon for the NAC species investigated in our current study.

In our current study of *C. parapsilosis* and *C. tropicalis*, malate synthase was also identified as a fibronectin, vitronectin and laminin binder. Interestingly, in another fungal pathogen, *Paracoccidioides brasiliensis*, this enzyme was shown to be exposed at the cell surface and to bind fibronectin, collagen type I and collagen type IV, and to also be responsible for adhesion of the fungus to pulmonary epithelial cells [49].

The above comparisons between three *Candida* species indicate that *C. albicans* and *C. parapsilosis* share some similar ECMP-binding proteins such as Als-like proteins, a protein similar to Hwp1 and phosphogluconate mutase. However, even for those two species additional proteins were identified, possibly accounting for slight differences in the ECMP-binding levels observed in this study. Interestingly, the comparisons between *C. tropicalis* and *C. albicans* ECMP-binding proteins, despite a similar adhesiveness of these two species, did not indicate any common ECMP binders. These differences can be taken into consideration to propose in the future some new strategies to reduce the ECM-binding ability of *C. tropicalis* that seems to rely on other molecules than those used by *C. albicans*. Such an approach might be useful for both a diversification and an extension of currently used therapeutic strategies, especially those based on the inhibition of the fungus interactions with host proteins and tissues.

## Conclusions

The results of our present study are both extensive and consistent, due to the use of two methods to extract the fungal cell wall-associated proteins and two methods (affinity chromatography and cross-linking) to qualify the ECMP-binding activity. Further detailed analysis will probably confirm the roles of individual proteins in ECM–fungal cell interactions. The collective action of all of the above-mentioned fungal ECMP binders should greatly enhance the ability of *C. parapsilosis* and *C. tropicalis* to adhere to the ECM and facilitate the invasion of human hosts. Detailed characteristics of this molecular pathogenicity mechanism for the investigated NAC species could add to the development of new methods for treating severe infection caused by these emerging pathogens.

## Abbreviations

AFC: affinity chromatography
BSA: bovine serum albumin
CL: cross-linking
DTT: dithiothreitol
ECM: extracellular matrix
ECMP: extracellular matrix protein
ESI: electrospray ionization
FN: fibronectin
LAM: laminin
LC: liquid chromatography
LC-MS/MS: liquid chromatography-coupled tandem mass spectrometry
MS: mass spectrometry
MS/MS: tandem mass spectrometry
NAC: non-albicans *Candida* species
SA-HRP: horseradish peroxidase-conjugated streptavidin
SDS: sodium dodecyl sulphate
SDS-PAGE: sodium dodecyl sulphate polyacrylamide gel electrophoresis
sulfo-SDAD: sulfosuccinimidyl 2-([4,4′-azipentanamido]ethyl)-1,3′-dithiopropionate
PBS: phosphate-buffered saline
UHPLC: ultrahigh-pressure liquid chromatography
VTN: vitronectin
